# 

**Published:** 1859-05

**Authors:** 


ORIGINAL COMMUNICATIONS.

PAGE.

ART. I. Notes of Experiments on the Effects of Woorara introduced into the Stomach. By Daniel Brainard, M.D., Professor of Surgery in the College et Chicago, Illinois.. 261
ART. II. Cases illustrating the Practical Use of the Opthalmoscope, as a means of Diagnosis in certain Diseases of the Eye. By E. L. Holmes. M.D., of Chicago, Ill... 266
ART. III. Foreign Bodies in the Air Passages. Case of Intestinal Concretion. By Charles Brackett, M.D., Rochester, Ind........ 271
ART. IV. Case of Ununited Fracture of the Radius of about twelve weeks' standing, cured by Sub-Cutaneous Perforation of the Bone. By A. L. McArthur, M.D., of Joliet, Ill........ 273
ART. V. Case of Protracted Gestation. Bv Dr. W R. Stone, of Manhattan, Ind...... 275

TRANSLATIONS FROM FOREIGN JOURNALS.

Two Memoirs on the Variations of Color of the Venous Blood. By Prof. Claude Bernard. Translated by Thos. Bevan, M.D., Chicago........ 277
Abstract of an Article on the "Anatomy and Physiology of the White Corpuscles of the Blood (Leucocytes)." By Dr. C. Robin. Translated by E. L. Holmes, M. D., Chicago........ 282
Resection of the Knee Joint Translated by the Editor........ 285
On the Artificial Production of Bone. By Dr. Leopold Ollier. Translated by E. L. Holmes, M.D........ 288

EXTRACTS.

Nursing Sore Mouth; its Nature, Cause, and Treatment. By T. J. W. Pray, M.D., Dover, N. H........ 291

BOOK AND PAMPHLET NOTICES.

Manual of Botany. By Asa Gray........ 314
First Lessons in Botany and Vegetable Physiology. By Asa Gray........ 314
Ventilation in American Dwellings. By D. B. Reid, M.D........ 318
Selections from Favorite Prescriptions of American Practitioners. By Horace Green, M. D........ 318
Five Essays. By John Kearsley Mitchell, M.D........ 319

EDITORIAL.

To our Patrons........ 320
Convention of Medical Teachers........ 320

MISCELLANEOUS MEDICAL INTELLIGENCE.

Surgical Matters—Ovariotomy........ 321
Meeting of the DeWitt County Medical Society........ 322
Medical Convention for revising the Pharmacopœia of the United States........ 323

